# Pancreatitis

**Published:** 1901-05-18

**Authors:** 


### PANCREATITIS.

Great strides have been made during the past year or two in our knowledge of diseases of the pancreas, especially in regard to the inflammatory affection of that organ. Among the most striking observations on the subject have been those made by Mayo Robson and communicated to the International Medical Congress in Paris last year, when he showed that many of the cases hitherto regarded as cancer of the head of the pancreas are really cases of chronic inflammation of that organ. In an address delivered before the American Surgical Association, Mr. Robson again deals with the subject of pancreatitis. He points out as a curious fact that although surgeons have been removing gall stones from the common duct for over a hundred years, and although the subject of jaundice dependent on obstructed common duct has received great attention from physicians for many years, yet until comparatively lately it seems never to have dawned upon the minds of clinical observers that whatever obstructs the common duct at its lower end must of necessity lead also to an obstruction in the pancreatic duct. Although infective and suppurative cholangitis has been well recognised by pathologists, yet infective and suppurative catarrh of the pancreatic ducts has even yet received no place in the medical text books. Yet we know, both by post-mortem and surgical experience, that under similar circumstances the pancreatic ducts participate in the same inflammatory processes as the bile ducts.

When the common bile duct is obstructed, the objective sign of jaundice at once demonstrates the fact; hitherto, however, no pathognomonic sign has been discovered which will show conclusively that the pancreatic ducts are occluded, unless it be extremely rapid loss of weight; but when it is borne in mind that the pancreatic duct opens along with the common bile duct into the second part of the duodenum, a channel usually containing septic organisms, it is not a matter for surprise that pancreatitis should be met with. As in the liver we may have simple, infective, and suppurating catarrh of the excretory ducts, as well as inflammation of the interlobular tissues, so in the pancreas we have similar diseases. The essential and immediate cause of the various forms of pancreatitis is bacterial infection induced by biliary and pancreatic lithiasis, injury, gastro-duodenal catarrh, ulcer and cancer of the stomach, pylorus or duodenum, and zymotic diseases, though in some cases pancreatitis has come on in persons in robust health, and the determining cause has been beyond recognition. Pancreatitis may be divided into acute, subacute, and chronic.

In acute pancreatitis, treatment practically resolves itself into that of peritonitis, commencing in the superior abdominal region. The pain at the onset is so severe as to necessitate the administration of morphine, and the collapse will probably demand stimulants, which may have to be given by enema. In the early stages the symptoms are usually so indefinite that the indications for surgical treatment are not clear enough to warrant operation, and until the collapse has passed off no surgical procedure would generally be justifiable. But so far as concerns the surgical aspect of the case an early evacuation of the septic matter is required, and an early exploration, from the front through the middle line above the umbilicus, or from behind through the left costovertebral angle, is demanded. After establishing the diagnosis by the anterior small incision and the introduction of a finger, the posterior incision, which must be a free vertical one in the left costo-vertebral angle, must be made so as to permit the insertion of the whole hand if thought necessary. This will enable the diseased organ to be very freely examined and if necessary drained for the evacuation of pus and gangrenous material, and from its position it will involve no risk to the general peritoneal cavity and will give opportunity for good drainage.

In subacute pancreatitis if surgical treatment is decided upon, a median incision above the umbilicus will enable the operator to locate any incipient collection of pus, which should then if practicable be evacuated by a posterior incision in the left or right costo-vertebral angle, or if this be thought impracticable it may be aspirated and he cavity opened and packed with gauze, which may be brought forward through a large rubber tube by which a track isolated from the general peritoneal cavity will soon be established. If a definite abscess form and approach the surface, in front or in either loin, the treatment will be incision and drainage as in the case of any other abdominal abscess.

Chronic pancreatitis must also be treated by abdominal section and drainage, but in this case the drainage is indirect and is obtained by draining the gall-bladder by cholecystotomy, or cholecyst-enterostomy, or duodeno-choledochotomy. It is best to begin by making a vertical incision through the upper part of the right rectus, splitting the muscle to whatever extent is necessary in order to obtain a good view of the diseased region, and when it has been decided what to do, which cannot be determined until the abdomen is opened, this incision may then be extended if necessary. It is important to remember how accurately malignant disease may be simulated by chronic interstial pancreatitis. This, says Mayo Robson, would make him hesitate to decline operation in any case of distended gall bladder where the patient was in a condition to bear it, or even in any case of chronic jaundice without distension of the gall bladder where the general health was deteriorating; as, though it should be recognised that if the disease should be really malignant very little good would be done, and life might even be shortened, yet if the disease should prove to be chronic pancreatitis a real and permanent cure might be brought about. The results of treatment in this class of cases have been most encouraging, and contrast markedly with those of the surgical treatment of cancer of the pancreas.

